# Airway hyperresponsiveness; smooth muscle as the principal actor

**DOI:** 10.12688/f1000research.7422.1

**Published:** 2016-03-09

**Authors:** Anne-Marie Lauzon, James G. Martin

**Affiliations:** 1 Meakins-Christie Laboratories, McGill University Health Center Research Institute, Montreal, QC, Canada; 2 Department of Medicine, McGill University, Montreal, QC, Canada

**Keywords:** Airway hyperresponsiveness, smooth muscle, Airway epithelium, asthma

## Abstract

Airway hyperresponsiveness (AHR) is a defining characteristic of asthma that refers to the capacity of the airways to undergo exaggerated narrowing in response to stimuli that do not result in comparable degrees of airway narrowing in healthy subjects. Airway smooth muscle (ASM) contraction mediates airway narrowing, but it remains uncertain as to whether the smooth muscle is intrinsically altered in asthmatic subjects or is responding abnormally as a result of the milieu in which it sits. ASM in the trachea or major bronchi does not differ in its contractile characteristics in asthmatics, but the more pertinent peripheral airways await complete exploration. The mass of ASM is increased in many but not all asthmatics and therefore cannot be a unifying hypothesis for AHR, although when increased in mass it may contribute to AHR. The inability of a deep breath to reverse or prevent bronchial narrowing in asthma may reflect an intrinsic difference in the mechanisms that lead to softening of contracted ASM when subjected to stretch. Cytokines such as interleukin-13 and tumor necrosis factor-α promote a more contractile ASM phenotype. The composition and increased stiffness of the matrix in which ASM is embedded promotes a more proliferative and pro-inflammatory ASM phenotype, but the expected dedifferentiation and loss of contractility have not been shown. Airway epithelium may drive ASM proliferation and/or molecular remodeling in ways that may lead to AHR. In conclusion, AHR is likely multifactorial in origin, reflecting the plasticity of ASM properties in the inflammatory environment of the asthmatic airway.

## Introduction

For decades, the excessive responses of the asthmatic airway to bronchoconstrictive stimuli have been recognized in association with asthma. Standardization of bronchial provocation testing with histamine initially and methacholine subsequently led to quantification of airway responsiveness (AHR) and ultimately its incorporation into the definition of asthma. Although loosely associated with the severity of asthma, the quantification of AHR in the pulmonary function laboratory provides a measure of the probability that the person studied suffers from some of the symptoms attributable to asthma. Despite its limited clinical usefulness, the assessment of AHR to challenge with inhaled methacholine triggered by stimuli such as allergens has been the principal surrogate for asthma in attempts to model the disease in animals. The environment-host interactions leading to AHR are multiple and the explanation for AHR is likely multifactorial. It is intuitively obvious that airway smooth muscle (ASM) is at the center of the problem, since it is the principal actor in airway narrowing. However, the extent to which the milieu in which it functions is responsible for excessive shortening of ASM or altered properties of ASM confer the excess responsiveness remains unclear ^1^. In addition, the expanded role of ASM in the biology of the airway has emerged as the synthetic capacity of ASM has been elucidated. This commentary reviews some of the proposed explanations for the occurrence of AHR with a particular focus on the ASM.

## Mechanical determinants of AHR

**Figure 1.**
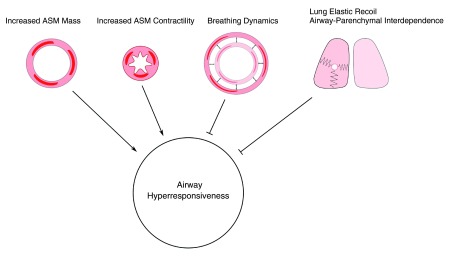
Mechanical determinants of airway narrowing. Airway narrowing is favored by enhanced contractility of airway smooth muscle (ASM) and by an increase in mass of ASM. Potent inhibitors of airway narrowing are lung elastic recoil, airway-parenchymal interdependence, and oscillatory stretches of the ASM caused by breathing movements and intermittent deep breaths.

Airway narrowing results from the effects of ASM contraction and has been thoroughly analyzed from the standpoint of its mechanical determinants. A static analysis of a model of the airway tree as a tube which constricts in response to contraction of ASM embedded in the wall has provided useful insights into the determinants of airway resistance and AHR ^2– 4^. The theoretical importance of geometric factors such as increase in area of tissue between the ASM and the lumen, the mass of ASM, and the elastic recoil of the parenchyma surrounding the airway was demonstrated. The exquisite sensitivity of airway responsiveness to lung volume *in vivo* ^5, 6^ supports the idea that lung elastic recoil is crucial in opposing the capacity of ASM to narrow the airways. However, a static force-balance analysis is incomplete. Interaction of contracting ASM with stresses imposed on the airway wall by breathing has demonstrated substantial effects on ASM behavior ( Figure 1).

The lack of a bronchodilating effect of a deep breath in asthma has been extensively documented and has prompted its further exploration as a fundamental characteristic of the asthmatic airway ^7^. The imposition of a tidal breathing pattern that precludes any deep breath during the inhalation of the bronchoconstrictor methacholine augments the degree of induced bronchoconstriction in non-asthmatic subjects ^8^. The reason for the failure of asthmatics to respond to a deep breath with bronchodilation remains uncertain. Stiffening of the asthmatic airway from remodeling ^9^ is one potential reason, since the lengthening of the ASM will be less for any given change in airway transmural pressure imposed by a breath. Observations on intact human subjects have thus far not resolved the fundamental issue of the root cause of AHR. Perhaps further study of tissues from human lungs will help to clarify.

## Contractile properties of ASM

### Intrinsic properties

ASM exhibits force-length characteristics reminiscent of skeletal muscle, although it retains the capacity to generate force over a much wider range and to shorten to much smaller lengths ^10^. The range of shortening suffices for complete airway closure, and so effective mechanisms to limit shortening are required, as discussed above. A particularly remarkable property of ASM is its ability to readjust its molecular motors when length changes are sustained so that it regains the ability to generate force ^11^. In other words, its force-length relationship is plastic and appears to be best explained by the addition or subtraction of myosin-containing contractile units in series to accommodate the new length ^12^. Additionally, the induction of increased tone in ASM results in an increase in force generated in response to a further contractile stimulus ^13^, suggesting that bronchoconstriction may beget bronchoconstriction. The maintenance of force by ASM may involve additional molecular mechanisms that affect actin-myosin interactions and potentially actin regulatory proteins such as calponin ^14^ and caldesmon ^15^. Cytoskeletal re-organization that may not involve the classical contractile apparatus of ASM may also affect force generation, a process that is sensitive to the inhibition of Rho kinase ^16^.

In recent years, much attention has been paid to the interaction of superimposed length changes on ASM that is concomitantly stimulated to contract. *In vitro* cyclical stretching of pre-contracted ASM causes the ASM to lose much of its tension ^17^. These oscillatory forces applied to the ASM are predicted to be equivalent to the stresses imposed by tidal breathing on the airways leading to a reduction of active force, described as fluidization. Whether tidal breathing exerts the predicted degrees of stretch of ASM *in vivo* that have been used *in vitro* has been questioned ^18^. Some of the impact of stretching of ASM may be offset by changes in the contractile apparatus that counteract the dilating effects of the imposed length changes ^19^. Chin and colleagues have found that tracheal smooth muscle harvested from asthmatic subjects shows a smaller reduction in force following imposed length changes compared to non-asthmatic tissues ^20^. Another study that failed to confirm these findings may have studied asthmatics of lesser severity ^21^. If severe asthma is associated with a resistance of contracted ASM to lose force with length change, then understanding this phenomenon will help us to understand the observed lack of bronchodilation in these subjects after a deep breath ^7, 8^. Currently, the softening of ASM is not completely explained but is attributed to disruption of either actin-myosin bridges or cytoskeletal proteins that contribute to the cellular stiffness. Fluidization is also sensitive to and enhanced by Rho kinase inhibition ^16^. This pathway may in the future provide therapeutic possibilities for the reversal of severe bronchoconstriction as witnessed during asthma attacks, where reversal by usual bronchodilator therapy is not observed.

It seems inconceivable that the ASM does not have a central role in AHR. Rats that show innate hyperresponsiveness to contractile agonists *in vivo* also show ASM hypercontractility at the level of tissue and cells ^22, 23^. These findings appear to be explained by differences in the metabolism of inositol trisphosphate ^24^, the principal mediator of calcium release within ASM cells. In contrast studies of the properties of ASM harvested from asthmatic subjects have, in general, failed to reveal hyperresponsiveness at the tissue level ^20, 21^, although peripheral airways have not been well studied. However, impaired relaxation has been observed and linked to β-adrenergic receptor uncoupling ^25^ and greater degradation of 3’,5’-cyclic adenosine monophosphate by phosphodiesterase ^26^. Ultrastructural studies of trachealis muscle have failed also to reveal differences between tissues harvested from asthmatic and non-asthmatic subjects ^27^. Biopsied ASM shows changes in the expression of a range of contractile proteins, compatible with molecular remodeling of the ASM in asthma ^28, 29^. However, not all studies report similar findings ^30^, although differences in the severity of the asthma may account for some of the discrepancies. In equine asthma (heaves), one of the few authentic models of asthma, the smooth muscle of the peripheral airways of the heaves-affected horses exhibited a greater unloaded shortening velocity (maximal velocity of contraction [Vmax]) compared to their own trachealis and compared to peripheral ASM of control horses ^31^. This supports the need for further studies of the more relevant intrapulmonary airways.

It is easily conceivable that an increase in ASM force generation, rather than an increase in the Vmax, may lead to AHR. An increase in ASM force is likely to override the impedance to airway narrowing resulting from lung elastic recoil and airway-parenchymal interdependence. The force, however, is difficult to quantify, as it depends on the stress generated by the ASM and the total amount of muscle and thus must be normalized by the cross-sectional area (stress) of ASM. Studies have so far shown no differences in stress between asthmatic and control airways ^20, 21^, but if the total amount of ASM is increased then the total force will increase too. This will require further mechanical investigations at the scale of the whole airway, preferably addressing the peripheral airways. The mechanism by which an increase in Vmax may lead to AHR is by counteracting the relaxing effect of tidal breaths ^1, 32^ by rapid recontraction. Studies using high-resolution computed tomography have shown that the airways from asthmatic subjects dilate upon stretching but that they quickly get back to their initial diameter ^33^.

### Induced properties

**Figure 2.**
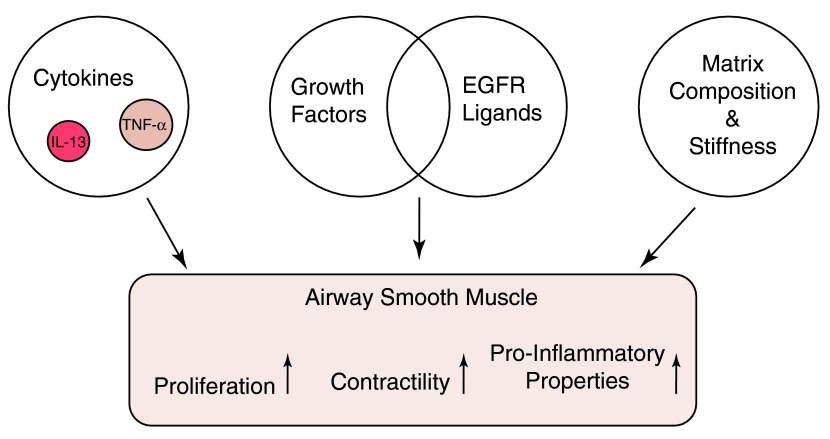
The modulation of airway smooth muscle properties by the asthmatic milieu. Airway smooth muscle (ASM) function is modulated by cytokines such as interleukin (IL)-13 and tumor necrosis factor (TNF)-α that increase intracellular calcium release in response to agonists such as histamine. ASM is triggered to proliferate *in vivo* by release of epidermal growth factor receptor (EGFR) ligands. The stiffness and composition of the matrix change ASM phenotype and may promote pro-inflammatory properties.

Alterations in ASM in response to the inflammatory environment represent another mechanism to account for AHR and one that may be distinct from innate differences in ASM properties ( Figure 2). Several cytokines involved in many cases of asthma such as interleukin (IL)-13 and tumor necrosis factor-α (TNF-α) alter the properties of ASM through effects on calcium signaling ^34^. IL-13 evokes a calcium signal in murine ASM cells and causes their contraction ^35^. Human ASM cells do not show this calcium spike following treatment with IL-13 but have enhanced calcium responses to stimulation by histamine ^36^. A series of studies has linked this finding to an upregulation of the bifunctional enzyme CD38 ^37^, which synthesizes cyclic adenosine diphosphate (ADP) ribose, the mediator of calcium release, via the ryanodine receptor. TNF-α appears to enhance calcium signaling by similar mechanisms ^38^. IL-13 additionally increases the sensitivity of the molecular motors to calcium by stimulating Rho kinase ^39^. IL-13 has also been found to reduce the relaxant response to ASM cells to β-adrenergic stimulation ^40^ and is the Th2 cytokine that has been most closely linked to AHR. The sensitization of ASM by immunoglobulin E (IgE) also leads to enhanced contractility and again by mechanisms involving IL-13 but by its autocrine effects on ASM ^41^. IL-13 also triggers oscillatory calcium currents ^42^, which may lead to transcriptional effects, and downregulates a regulator of G-proteins, RGS2, leading to AHR in mice ^43^.

Contact of ASM with immune cells has also been shown to enhance the contractility of smooth muscle. Several studies have implicated T lymphocytes in the phenomenon ^41, 44^. The effect of T cells on ASM contractility has been linked to autocrine production of IL-5 and IL-1β. Incubation of rat tracheal rings with CD4 ^+ ^T cells leads to an increased Vmax along with alterations in contractile protein expression ^45^. These changes necessitate contact between the muscle and the T cells, suggesting that intercellular cell adhesion mechanisms are involved. Mast cells within the ASM bundles express IL-13 ^46^ and may also be a cellular mechanism by which contractility of ASM is altered in asthma.

## Proliferative/secretory properties of ASM

ASM may exist in different states, contractile or secretory/proliferative, and ASM cells harvested from asthmatic subjects differ in these states from those of non-asthmatic subjects, retaining characteristics across multiple passages in cell culture. Asthmatic ASM proliferates more readily, a characteristic that has been attributed to increased mitochondrial mass that is in turn related to calcium handling ^47^. This abnormality, which is conserved *in vitro*, is presumably nonetheless an induced property. Alterations in the transcriptional regulation of secreted molecules such as CXCL8 and synthesis of enzymes such as cycolooxygenase-2 by ASM cells are now being reported ^48– 50^ and may provide clues to the altered states of asthmatic ASM. The profile of matrix protein production by asthmatic and non-asthmatic ASM differs ^51^ with the possibility of altering the milieu in terms of its potential to support an inflammatory environment or to affect other aspects of ASM phenotype.

## Changes in ASM mass

For almost 100 years, it has been noted that ASM is present in increased quantities in the airways of asthmatics. Formal morphometric studies in the 1960s documented the changes in a quantitative manner ^52^. Given the abovementioned issues of the balance of force offsetting ASM shortening, the increase in mass has the potential to account for AHR ^3^. Such a conclusion hinges on whether the contractile properties of the ASM are unaltered by its growth. *In vitro*, such states are generally reciprocally regulated so that proliferation of the ASM is anticipated to result in a reduction in contractile proteins ^53^. Whether such a phenomenon occurs *in vivo* awaits demonstration. One such study suggested changes in proliferating trachealis ^54^, but a subsequent study found no decline in myosin heavy chain despite significant proliferation of cells within the tissue studied ^55^. In human asthmatic tissues, contractile cells show a substantial variation in the intensity of staining for smooth muscle-specific α-actin, suggesting cells with a range of contractile properties exist *in vivo* ^56^. Those staining poorly for α-actin were not well incorporated into smooth muscle bundles but were arranged loosely and without any obvious relationship to existing ASM architecture. These cells may have been myofibroblasts, long argued to be precursors of ASM. The orientation of ASM within the airway wall may have a profound influence on the degree of airway narrowing that follows from the ASM contraction ^57^. There is currently no evidence that addresses this latter issue.

Increase in ASM mass results predominantly from an increase in the number of cells ^58^. In some cases, hypertrophy contributes to the change. Several studies have observed proliferating cells among the ASM bundles, suggesting measureable turnover of the ASM tissue ^56, 59^. A corollary is that remodeling of the ASM is dynamic and therefore potentially reversible, although no studies in humans have reported such a phenomenon. Only modest changes in AHR result from corticosteroid treatment of asthma, suggesting that remodeling may be irreversible, assuming it accounts for AHR in the first place. However, in equine asthma, partial reversibility of increased ASM mass has been shown with antigen avoidance or corticosteroid treatment ^60^.

If the mass of ASM is increasing, what factors are responsible for the increase? Many studies have been performed on animal models showing that ASM remodeling can be inhibited by attenuating airway inflammation, a finding that is hardly surprising, although purely mechanical factors related to compression of the airway epithelium by bronchoconstriction may release potent growth factors such as heparin-binding epidermal growth factor-like growth factor (HB-EGF) ^61^. Other studies also implicate the airway epithelium as a potential source of modulators of ASM properties. The cysteinyl leukotriene LTD _4_ releases HB-EGF from airway epithelial cells ^62^, as does IL-13 ^63^. HB-EGF will not only cause ASM proliferation but also promote goblet cell differentiation. The EGF receptor and cysteinyl leukotrienes have both been implicated in ASM and goblet cell differentiation induced by repeated allergen challenge in murine models ^64^. Airway epithelial cells in co-culture exert a pro-proliferative effect on ASM cells, in part mediated by IL-6, IL-8, and monocyte chemoattractant protein-1 ^65^. ASM from asthmatic subjects proliferates more readily than non-asthmatic ASM in response to the stimulation of airway epithelium by house dust mite and involves the activation of protease-activated receptor-2, epithelial synthesis of cysteinyl-leukotriene, and increased expression of the cysteinyl-leukotriene-1 receptor ^66^.

Over-expression of platelet-derived growth factor by airway epithelial cells may cause ASM growth in mice and is elevated in the bronchoalveolar lavage of mice after allergen challenges ^67^, but whether it is involved in ASM remodeling caused by allergen is still unclear. Translation of the above findings to human asthma is yet to be done.

## Matrix and ASM properties

Matrix composition and stiffness both influence the programming of cells, including those of the ASM. ASM cells cultured on collagen-conjugated polyacrylamide hydrogels of varying elastic moduli increase cellular secretion of vascular endothelial growth factor (VEGF) ^68^. Stiff gels stimulate cell proliferation, reduce VEGF secretion, and reduce agonist-induced calcium responses of ASM cells ^68^. However, *in vitro* studies demonstrate that susceptibility of ASM growth to inhibition by corticosteroids is affected also by its culture upon collagen I, whereas inhibiting the binding to collagen via α2β integrin restores the sensitivity to corticosteroids ^69^. *In vivo*, the amount of collagen III and laminin in the ASM correlated with reversibility of the forced expiratory volume in 1 second (FEV _1_) ^70^.

## Conclusions

AHR in the asthmatic subject remains poorly explained. However, the behavior of ASM in the dynamic circumstances of bronchoconstriction *in vivo* may prove to be the cause of its excessive narrowing if the failure of usual mechanisms limiting bronchoconstriction occurs in asthma. Accelerated rates of contraction, impaired relaxation, and blunted relaxant responses to tidal breathing and deep inspirations are among the potential characteristics that might lead to greater airway narrowing. Stiffening of ASM because of cytoskeletal re-organization could conceivably fix the airway in a narrowed state, relatively refractory to relaxant agonists, as is observed in acute asthma exacerbations. Reprogramming of ASM seems to occur, or perhaps selective overgrowth of ASM with a greater capacity for proliferation and pro-inflammatory effects occurs. Such reprogramming might be expected to lead to ASM that is less contractile but has not been shown. The role that altered airway mechanics or epithelial effects plays in the ASM program may require further exploration.

## Abbreviations

ASM, airway smooth muscle; AHR, airway hyperresponsiveness; EGFR, epidermal growth factor receptor; HB-EGF, heparin-binding epidermal growth factor-like growth factor; IL, interleukin; TNF-α, tumor necrosis factor-α; Vmax, maximal velocity of contraction.
